# Depth of Anesthesia as a Risk Factor for Perioperative Morbidity

**DOI:** 10.1155/2015/829151

**Published:** 2015-06-02

**Authors:** Argyro Petsiti, Vassilios Tassoudis, George Vretzakis, Dimitrios Zacharoulis, Konstantinos Tepetes, Georgia Ganeli, Menelaos Karanikolas

**Affiliations:** ^1^Department of Anesthesiology, University of Larissa, Larissa, Greece; ^2^Department of Surgery, University of Larissa, Larissa, Greece; ^3^Department of Anesthesiology, Washington University School of Medicine, 660 S. Euclid Avenue, Campus Box 8054, St. Louis, MO 63110, USA

## Abstract

*Introduction*. The prognostic value of age, physical status, and duration of surgery on perioperative course has been extensively studied. However, the impact of deep hypnotic time (time when Bispectral Index values are less than 40) has not been well evaluated. *Methods*. We designed an observational study to clarify the relative influence of deep hypnotic time (DHT) on outcome. Eligible participants were mentally stable patients over 18 years old scheduled for elective major abdominal surgery. In total, 248 patients enrolled. Data were analyzed using Fisher's exact test and multiple logistic regression. *Results*. Five variables (DHT, hypotension, age, comorbidity, and duration of surgery) showed statistically significant association with complications, when examined independently. However, when all variables were examined together in a multiple logistic regression model, age and comorbidity were no longer associated with outcome. DHT, hypotension, and duration of surgery were significant predictors of “complications,” and “hypotension” was a significant predictor of prolonged hospital stay (*P* < 0.001).  *Conclusion*. Deep hypnotic time emerged as a new factor associated with outcome, and its impact compared to other factors such as age, surgery duration, hypotension, and comorbidity is redefined. Monitoring and managing depth of anesthesia during surgery are important and should be part of careful operation planning.

## 1. Introduction

The role of physical status and age on patient outcome after major surgery has been thoroughly studied [1]. It is well established that patient condition at the time of hospital admission as defined by the American Society of Anesthesiologists (ASA) Physical Status or the Charlson comorbidity score [2] correlates closely with postoperative mortality, which can be as high as 10% in elderly patients, particularly when they undergo urgent surgery [3–6]. Duration of surgery has also been shown as important factor significantly affecting outcome [7, 8]. With regard to anesthetic management, published reports suggest that the risk of morbidity and mortality associated with anesthesia is extremely low [9].

In 2005, an innovative study by Monk et al. attempted to identify new variables such as duration of deep hypnosis defined as low Bispectral Index values and duration of intraoperative hypotension as potential risk factors for long-term (one-year) postoperative mortality [10]. These authors reported that the relative risk of mortality for deep hypnotic time (DHT, defined as the cumulative period of Bispectral Index values < 45) and intraoperative systolic hypotension was 1.244/hour and 1.036/minute, respectively. Furthermore, another study reported relative risk of 1.244/hour concerning BIS values under 45, but the authors could not ascertain whether this finding was causal or coincidental [11]. As expected, these findings provoked discussion and criticism in the scientific community, since several other factors could contribute to mortality during long study periods. In our study, the observation period was limited to the time during which patients were hospitalized, thus avoiding the impact of coincidental factors on morbidity and mortality.

In a previous study, we demonstrated that persistent hypotension during surgery is a significant risk factor for postoperative complications [12]. In addition, data from the B-Aware study have shown that the risk of myocardial infarction, stroke, and death was higher in patients with intraoperative BIS values < 40 for more than 5 minutes [13]. Because intraoperative hypotension may be related to deep anesthesia, the possible association between deep anesthesia and hypotension raises a question about the role of deep anesthesia on postoperative complications. The above concerns regarding the association between hypotension and mortality motivated us to reevaluate the goals of anesthetic management. In this prospective observational study we attempted to investigate factors such as DHT and hypotension, which could alter clinical course while in hospital and could therefore be associated with long-term outcomes.

## 2. Methods

Following approval by the Institution Ethics Committee, we conducted this prospective observational study at a tertiary care university hospital. The study included patients scheduled for major elective abdominal surgery (expected duration > 2 hours) over a two-year period. Inclusion criteria were mentally healthy patients who had given written informed consent and elective major abdominal surgery. Exclusion criteria were pregnancy, inability to comprehend or follow orders, history of psychiatric disease or mental instability, and use of drugs that could influence the central nervous system. Demographic and clinical data for every patient were entered in a secure electronic database. Variables recorded included age, gender, marital status, educational level, alcohol and tobacco use, body mass index (BMI), and patient physical status characteristics. Physical status was evaluated using three widely used methods: the New York Heart Association (NYHA) Functional Class, the ASA Physical Status, and the Charlson Comorbidity Index in order to establish comorbidity scores on admission to hospital. All data were collected by trained physicians and were reviewed for reliability and completeness immediately after collection.

In the operating room, monitoring included five lead ECG and continuous blood pressure monitoring using a radial arterial line. Before induction of anesthesia, a low thoracic (T9–T12) epidural catheter was inserted and was used for perioperative analgesia, with incremental administration of 0.5–1.5 mL/dermatome (depending on patient age) of a preservative-free solution containing ropivacaine 3.75 mg/mL and fentanyl 2 mcg/mL. Anesthesia was induced with 100 mcg fentanyl, 1-2 mg midazolam, and 1.5–2 mg/kg propofol and was maintained with 1.1–1.2 MAC sevoflurane. Cisatracurium was used to facilitate tracheal intubation and maintain neuromuscular blockade. A Bispectral Index monitor was used to measure depth of anesthesia (A1050 BIS Monitor and BIS sensor; Aspect Medical Systems, now part of Covidien). In the recovery room, a programmable infusion pump was used to administer a mixture of ropivacaine 1 mg/mL and fentanyl 2 mcg/mL for patient-controlled epidural analgesia, using the following settings: basal infusion 7 mL/hour, bolus 3 mL, and lockout time 30 minutes. The postoperative analgesic protocol also included intravenous paracetamol 600 mg every 6 hours and intravenous parecoxib 40 mg twice a day.

Perioperative data, including surgery type and duration, anesthetic drugs used (including total dose of local anesthetic), hemodynamic variables, fluid and blood product administration, and urine output were recorded on a standardized data collection sheet. BIS values were automatically transferred to an electronic database and were then transferred to the main database. Recording was meticulously inspected for artifacts, such as high BIS values with muscle activity, and all artifacts or obviously erroneous readings were corrected manually. The summation of time with BIS values lower than 40 was recorded as total DHT in minutes. We chose to use BIS < 40 as cutoff value for DHT, because 40 has been used as cutoff value in clinical studies, including the B-Aware trial, has been shown to be associated with morbidity and mortality [13], and is the lower end of the range recommended by the BIS manufacturer.

Patients were assigned to two groups based on intraoperative DHT: (a) patients with DHT = 0 and (b) patients with DHT > 0. Hypotension was defined as mean arterial pressure (MAP) < 60 mm Hg or as MAP < 70 if MAP had decreased by more than 30% below baseline [14]. The summation of low values was recorded as total hypotension time (THT, in minutes). Because brief periods of hypotension are common in almost every operation [15], patients were allocated to two groups based on intraoperative THT: (1) THT ≤ 10 minutes and (2) THT > 10 minutes for the entire operation.

Patients were evaluated daily until death or hospital discharge. Postoperative data were recorded on standardized data sheets and included pain assessed with visual analog scale, additional analgesic requirements, patient sedation, nausea or vomiting, itching, mobilization, oral intake of liquids or food, epidural catheter removal, medications administered or skipped, sleep disturbances, and complications. All patients were assessed by interview, clinical examination, medical record review, and consultation with their primary in-hospital healthcare provider. For the purposes of this study, complications were defined as the need for any acute medical intervention or treatment. Patients were divided into two groups depending on whether or not they had postoperative complications. The scope of complications was not restricted to major complications but also included lesser forms of morbidity which can influence length of hospital stay and healthcare cost.

For the purposes of data analysis, we treated gender as a qualitative characteristic and defined clear cutoff points for all other variables that were analyzed. The cutoff point for age was 65 years, since 65 is the retirement age in most Western societies and also because morbidity and mortality increase with age > 65 [10, 16]. With regard to comorbidity, patients were divided into two groups: those with Charlson score ≤ 2 and those with Charlson score > 2. The Charlson comorbidity score was selected because it seems superior to other methods such as the ASA Physical Status; a Charlson score > 2 is associated with 16-fold increased risk of 1-year mortality and therefore is considered a better prognostic index [2, 10]. Similarly, patients were divided into two groups based on duration of surgery, using a cutoff of 180 minutes (standard duration when surgery lasted ≤ 180 minutes versus long duration if surgery lasted > 180 minutes). A retrospective analysis of data from patients who had major surgery in our hospital showed that patients with few complications stayed up to 9 days (maximum); therefore, the cutoff point for long hospital stay was 9 days. Furthermore, patients were assigned to one of two groups based on alcohol use and on tobacco use.

Statistical analysis was performed using the SPSS version 17.0 statistical software package (SPSS Inc., Chicago, IL). For the purposes of this analysis, patients were divided into two groups for each independent variable (age, gender, tobacco use, duration of surgery, complications, and hospital stay) using clear cutoff points, as described above. Two variables (“complications” and “length of hospital stay”) were assessed as dependent variables. Categorical variables were compared between groups using Pearson's chi square or Fisher's exact test. Multivariate logistic regression was then used to analyze all variables together and adjust the specific risk that each independent variable attributed to the dependent variable. In a univariate basis, the (unadjusted) risk from any independent variable could be attributable to some other independent variable, if an interaction existed. Therefore, all variables (surgery duration, hypotension, Charlson comorbidity score, age, and DHT) were analyzed together using logistic regression in order to improve prediction accuracy for the dependent variable. The Hosmer-Lemeshow goodness of fit test was used to assess how well each logistic regression model fits the data. In addition, collinearity diagnostics (Eigenvalues and Condition Indexes, calculated in SPSS) were used in an attempt to assess if collinearity between predictor variables was a concern for the regression models. Observed associations are expressed as adjusted odds ratio (OR) with 95% confidence intervals (CI). An effect was considered significant when *P* < 0.05.

## 3. Results

In the 2-year study period 248 consecutive patients (120 women, 128 men) enrolled. Demographic and clinical data are summarized in Tables 1 and 2. 67 patients used tobacco and 87 used alcohol. Mean patient age was 63.8 ± 11.2 years. Surgery duration was 232 ± 55.3 min and length of hospital stay was 10.8 ± 7.3 days, while mean DHT was 28.7 ± 4.14 minutes and hypotension time was 23.5 ± 32.8 minutes. Patients could experience more than one event defined as “complication” during hospitalization.

Five variables (age, surgery duration, hypotension, DHT, and Charlson comorbidity score) showed significant association with “complications” by Fisher's exact test (Table 3). Of all these variables, only hypotension and complications showed statistical significance and related strongly to “hospital stay.” ASA Physical Status, NYHA classification, and gender did not show significant association with complications. Similarly, the variables “level of education,” “alcohol use,” and “tobacco use” did not show any significant effect.

The five variables found to be significantly associated with “complications” were analyzed further, using two multivariate logistic regression models.

In the first model, “complications” was the dependent variable, whereas in the second model “hospital stay” was the dependent variable. In both models the Hosmer and Lemeshow test for goodness of fit showed *P* > 0.05 (0.805 and 0.384, resp.), thus confirming that observed and expected rates were well calibrated and the models were well adjusted. When we corrected (adjusted) the influence of each factor by entering all variables into the multivariate logistic regression model (Table 4), the variables “age” and “Charlson score” did not show statistical significance (*P* = 0.191 and *P* = 0.232, resp.), thereby implying that the variance of the dependent variable “complications” could be attributed to factors such as surgery duration, hypotension, or DHT. The risk of complications increased 4.170-fold (95% confidence interval 1.079–16.117, *P* = 0.038) if a patient was under deep anesthesia, increased 2.946-fold (95% CI 1.117–7.773, *P* = 0.029) with lengthy surgery, and increased 4.670-fold (95% CI 1.744–12.508, *P* = 0.002) with intraoperative hypotension. A higher percentage of patients under deep anesthesia developed complications (56%) compared to patients not under deep anesthesia (5.2%). Furthermore, 36% of patients with any complication stayed longer in hospital compared to those who had none (0.8%).

In the second model, “hospital stay” was analyzed as dependent variable. When the variable “complications” was entered in the analysis as independent variable together with all other variables, it was the only variable that remained significant (*P* < 0.001). However, when the variable “complications” was excluded from the model, because of concerns about multicollinearity, “hypotension” was the only significant predictive variable (Table 5), so that patients with hypotension had a 4.269-fold (95% CI 1.743–10.455, *P* < 0.001) increased risk of prolonged hospital stay.

## 4. Discussion

This is a prospective observational study evaluating factors that could be associated with increased morbidity and mortality. Anesthetic depth emerged as a new factor, and its impact on outcome is redefined compared to other factors such as age, surgery duration, and comorbidity.

The observation period was limited to hospital stay, as we assumed that additional external risk factors could intervene and make it difficult to reach valid conclusions regarding morbidity and mortality after discharge from the hospital. Assuming that certain preexisting factors could influence outcome, the important question was which variables should be selected as potential predictors of poor outcome. This question is not easy to answer, because several interrelated factors may contribute to poor outcome, and although addition of new variables into the analysis might produce more accurate results, it could also increase the risk of producing spurious findings if too many variables were involved [17]. Another reason for avoiding full multifactorial analysis is the fact that several factors are not modifiable; therefore, little or nothing can be done about them. In that case, the analysis would provide information of limited practical value, instead of serving as a useful investigation of modifiable factors that could influence outcome. Therefore, although factors such as sepsis, lung disease (chronic obstructive pulmonary disease), and left ventricular injection fraction < 15% were recorded, they were not included in the analysis. We analyzed data with binary logistic regression, in addition to Fisher's exact test, in an attempt to eliminate confounding factors. While designing the study, considerable thought was given to selecting a limited number of appropriate, useful variables, with selection guided by common sense, clinical experience, and the literature. After taking into account the fact that factors reported in the literature as important have puzzled experts and it is not clear whether they influence outcome and how their influence could be modified, we selected the variables age, comorbidity, DHT, hypotension time and duration of surgery for the final analysis.

Using Fisher's exact test, our results are in agreement with earlier studies, which identified age, comorbidity, and surgery duration as factors influencing postoperative outcome. With regard to DHT and hypotension time, little is known about their significance, but our findings are consistent with findings of previous studies showing that DHT is associated with increased one-year mortality [10, 11]. In contrast, the variables “gender,” “alcohol use,” and “tobacco use” did not correlate with poor outcomes, possibly because the sample was too small to detect their effect.

Multiple logistic regression was employed to analyze all relevant factors simultaneously and showed that age and Charlson score no longer reached statistical significance, while duration of surgery, hypotension time, and DHT remained significant, thus implying a close relation with complications. This observation is important and suggests that physicians should probably pay close attention not only to patient age and physical status but also to anesthetic management, including DHT and intraoperative hypotension. In addition, efforts aimed at limiting duration of surgery are probably worthwhile.

After the first part of the analysis was completed, a second analysis, with “hospital stay” as dependent variable, showed that “hypotension time” was associated not only with “complications” but with “hospital stay” as well. This finding suggests that intraoperative hypotension may be truly important, as it is associated with both the risk of complications and the probability of prolonged hospital stay.

The implications of this study are that anesthetic management may contribute to poor outcome; therefore, new approaches to improving outcome should be considered. This is particularly true with DHT, because it is the only easily modifiable factor considered here, whereas age cannot be modified and physical condition or surgery duration is difficult to change. Hypotension is also important but despite physician attention to blood pressure control and efforts to avoid hypotension, this goal can be difficult to achieve during major surgery. It is well known that anesthesia, surgery, and postoperative pain are strong interacting elements and may alter the cytokine (TNF and interleukin) profile. Inflammatory response plays a major role in tissue repair and patient healing and can lead to immune system suppression, hypoperfusion, coagulopathy, and complications such as infection, ischemia, multiple organ failure, sepsis, or death [18–24]. It has been suggested that controlling anesthetic depth affects postoperative pain levels and reduces postoperative analgesic consumption [25, 26], but this finding has been questioned by other studies with contradictory findings [27]. Since opiates were used for postoperative analgesia in these studies, it is not clear how deep anesthesia with low BIS values could impact the severity of postoperative inflammation when perioperative analgesia is provided with epidural analgesics. In our study we chose thoracic epidural analgesia for intra- and postoperative pain control, because epidural analgesia has been shown to control the catabolic response to surgical stress, even though it has not been shown to affect inflammation markers or hospital stay [28–31].

Pulmonary and gastrointestinal dysfunction rather than cardiac complications were the most important complications in our study (Table 2), whereas other studies identified cardiac events as the main cause of morbidity after noncardiac surgery [32]. This difference could be due to the fact that, in contrast to studies evaluating specific operations, such as vascular surgery, patients in our study underwent a variety of abdominal operations. In addition, we recorded all major and minor complications, whereas other studies focused solely on serious events, such as myocardial infarction, and therefore may have underreported lesser complications. The gastrointestinal dysfunction observed in our report is consistent with other studies showing that the gastrointestinal tract is extremely sensitive to hypoperfusion and to the release of catecholamines and inflammatory mediators [33, 34]. Although surgical trauma in abdominal surgery profoundly contributes to gastrointestinal dysfunction, another report showed that 32% of patients had gastrointestinal dysfunction after extra-abdominal operations, such as hip arthroplasty [35]. Considering that anesthetic drugs and techniques can influence the inflammatory response, our findings suggest that prolonged surgery, prolonged deep anesthesia, and intraoperative hypotension may exacerbate the stress response and increase the number of complications, thereby raising morbidity and worsening outcomes, including, perhaps, increased one- to two-year mortality [10, 11].

Limitations of this study include study design, as observational studies are generally considered less rigorous than prospective clinical trials. In addition, it is possible that our sample may not be representative of the population and the sample size may not be large enough to show significant effects [17]. We attempted to keep the sample random, by avoiding preselection method, other than the exclusion criteria listed in Section 2. It should also be emphasized that anesthetic and surgical teams remained the same throughout the study and all operations were elective, open abdominal, long duration procedures, in an attempt to minimize heterogeneity related to variability of surgical or anesthetic technique or individual physician skills. Lastly, selection of appropriate independent variables that could contribute to variance in “complications” or “hospital stay” is crucial in multivariate logistic regression, because introduction of additional independent variables in the analysis can alter the results and their interpretation. It is possible that our findings are result of random variability, but other studies [10, 11] strongly refute this hypothesis.

In conclusion, our study suggests that complications are common after elective major abdominal surgery, are related to underlying pathology and surgical factors, and are influenced by anesthetic management. Persistent low BIS values and prolonged hypotension are associated with bad outcomes but may be masked by confounding factors such as age or physical status. Considering the limitations of our study, well-designed prospective randomized trials are needed to better evaluate the impact of DHT and determine whether maintaining a lighter state of anesthesia can improve outcome.

## Figures and Tables

**Table 1:** Demographic and clinical characteristics.

*Demographic data *	
Age	63.8 (11.2) years
Gender (F/M)	120/128
BMI	25.7 (3.6)
Alcohol use	87
Tobacco use	67
*Medical history *	
Previous anesthesia	127
Under medication	170
Hypertension	117
Angina pectoris/myocardial infarction	20
Other heart diseases	37
Renal disease	7
Pulmonary disease	13
Stroke	2
Insulin-dependent diabetes	32
NYHA score >2	55
ASA Physical Status >2	80
Charlson comorbidity score >2	123

Parentheses denote standard deviation.

**Table 2:** Intraoperative and postoperative data.

Deep hypnotic time	28.7 (4.14) min
Surgery duration	232 (55.3) min
Hypotension time	23.5 (32.8)
Intraoperative ropivacaine use	78 (30.5) mg
Packed red cell transfusions	1.67 (0.765) units
Hospital stay	10.8 (7.3) days
Postoperative epidural analgesia	2.83 (0.95) days
Sleep disturbances	103
Nausea	1.2 (0.8) days
Vomiting	0.9 (0.7) days
Immobilized in bed or sitting	1.9 (2.1) days
Immobilization	2.2 (1.3) days
Fasting	2.2 (2.0) days
Oral liquids	1.9 (1.3) days
Parenteral nutrition	1.8 (3.1) days
Pulmonary complications (*n*, patients)	57
Cardiovascular complications (*n*, patients)	32
Gastrointestinal complications (*n*, patients)	51
Wound complications (*n*, patients)	25
Other complications (*n*, patients)	23
Postoperative ICU (*n*, patients)	4
Death (*n*, patients)	2

Parentheses denote standard deviation.

**Table 3:** Significance of associations with Fisher's exact test. Listed numbers indicate *P* values for each association. Associations are statistically significant when *P* < 0.05.

	Complications	Hospital stay
Surgery duration	0.013	0.539
DHT	0.011	0.157
Hypotension	0.000	0.000
Charlson comorbidity score	0.024	0.410
Age	0.040	0.408
Gender	0.542	0.835
Alcohol use	0.990	0.383
Tobacco use	0.645	0.640
Education	0.462	0.213
NYHA score	0.458	0.438
ASA Physical Status	0.086	0.827
Complications		0.000

**Table 4:** Multivariate logistic regression with “complications” as dependent variable.

	Adjusted OR	95% CI for OR	*P*
Hosmer and Lemeshow test			0.805
Age	2.058	0.698–6.068	0.191
Charlson score	1.924	0.658–5.631	0.232
DHT	4.170	1.079–16.117	0.038
Hypotension	4.670	1.744–12.508	0.002
Surgery duration	2.946	1.117–7.773	0.029

**Table 5:** Multivariate logistic regression with “hospital stay” as dependent variable.

	Adjusted OR	95% CI for OR	*P*
Hosmer and Lemeshow test			0.384
Age	0.583	0.484–3.639	0.583
Charlson score	1.085	0.397–2.967	0.874
DHT	2.514	0.600–10.525	0.207
Hypotension	4.269	1.743–10.455	0.001
Surgery duration	1.100	0.455–2.661	0.832
